# A large outbreak of Hepatitis E virus genotype 1 infection in an urban setting in Chad likely linked to household level transmission factors, 2016-2017

**DOI:** 10.1371/journal.pone.0188240

**Published:** 2017-11-27

**Authors:** Alexander Spina, Annick Lenglet, David Beversluis, Marja de Jong, Larissa Vernier, Craig Spencer, Fred Andayi, Charity Kamau, Simone Vollmer, Boris Hogema, Andrea Irwin, Roger Ngueremi Yary, Açyl Mahamat Ali, Ali Moussa, Prince Alfani, Sibylle Sang

**Affiliations:** 1 Médecins Sans Frontières, Operational Center Amsterdam (OCA), Ndjamena, Chad; 2 European Programme for Intervention Epidemiology Training (EPIET), European Centre for Disease Prevention and Control, (ECDC), Stockholm, Sweden; 3 Department for Infectious Disease Epidemiology and Surveillance, Austrian Agency for Health and Food Safety, Vienna, Austria; 4 Médecins Sans Frontières, Operational Center Amsterdam (OCA), Amsterdam, The Netherlands; 5 Department of Virology, Sanquin Blood Supply, Amsterdam, the Netherlands; 6 Ministère de la Santé Publique, N’djamena, Chad; Centers for Disease Control and Prevention, UNITED STATES

## Abstract

**Background:**

In September 2016, three acutely jaundiced (AJS) pregnant women were admitted to Am Timan Hospital, eastern Chad. We described the outbreak and conducted a case test-negative study to identify risk factors for this genotype of HEV in an acute outbreak setting.

**Methods:**

Active case finding using a community based surveillance network identified suspected AJS cases. Pregnant or visibly ill AJS cases presenting at hospital were tested with Assure^®^ IgM HEV rapid diagnostic tests (RDTs) and some with Polymerase Chain Reaction (PCR) in Amsterdam; confirmed cases were RDT-positive and controls were RDT-negative. All answered questions around: demographics, household makeup, area of residence, handwashing practices, water collection behaviour and clinical presentation. We calculated unadjusted odds ratios (ORs) and 95% confidence intervals (95% CI).

**Results:**

Between September and April 2017, 1443 AJS cases (1293 confirmed) were detected in the town(attack rate: 2%; estimated 65,000 population). PCR testing confirmed HEV genotype 1e.

HEV RDTs were used for 250 AJS cases; 100 (40%) were confirmed. Risk factors for HEV infection, included: having at least two children under the age of 5 years (OR 2.1, 95%CI 1.1–4.3), having another household member with jaundice (OR 2.4, 95%CI 0.90–6.3) and, with borderline significance, living in the neighbourhoods of Riad (OR 3.8, 95%CI 1.0–1.8) or Ridina (OR 3.3, 95%CI 1.0–12.6). Cases were more likely to present with vomiting (OR 3.2, 9%CI 1.4–7.9) than controls; possibly due to selection bias. Cases were non-significantly less likely to report always washing hands before meals compared with controls (OR 0.33, 95%CI 0.1–1.1).

**Discussion:**

Our study suggests household factors and area of residence (possibly linked to access to water and sanitation) play a role in HEV transmission; which could inform future outbreak responses. Ongoing sero-prevalence studies will elucidate more aspects of transmission dynamics of this virus with genotype 1e.

## Background

Studies between 1990–2013 estimate the global burden of Hepatitis E virus (HEV) infection around 3.4 million cases of symptomatic illness, 70,000 deaths and 3000 stillbirth on an annual basis [1]. The disease is usually self-limiting but in severe cases can develop into fulminant hepatitis. Five genotypes are known to infect humans. HEV genotype 3 and 4 are zoonotic and infect a large range of hosts with pigs probably serving as the major reservoir. Genotype 1 and 2 infect only humans and are spread by faecal contamination of water supplies, but household transmission has been shown to play a role [2]. Most recently, a case of post-transplantation HEV infection with genotype 7 was reported, which had only been known to be associated with camels prior to this case [3]. The overall case fatality rate is estimated between 4–30%, but has been documented to reach up to 40% in pregnant women, particularly during the third trimester [4–6].

Outbreaks of HEV infection have been reported in sub-Saharan Africa, with the majority of documented experiences in outbreaks centralised in camps of refugees or internally displaced persons in Kenya, Uganda, Sudan, South Sudan, Ethiopia and Chad [6–10]. HEV outbreaks in Chad were reported in 1983–1984 in French soldiers and refugees in Goz Amer in 2004 [6,11]. The infection appears to be common in the country with 22% of hospitalised patients for non-hepatic causes displaying a history of infection [12].

In epidemiological week 33, 2016 (September), three females patients were admitted to the hospital in Am Timan (managed collaboratively between the Ministry of Health (MoH) of Chad and Médecins Sans Frontières [MSF]) with acute jaundice syndrome (AJS). Of these, two died (both pregnant) from fulminant hepatitis and one tested positive for HEV infection using the Assure^®^ IgM HEV Rapid Diagnostic test (RDT) [13]. Am Timan is a town of approximately 65,000 persons in the eastern Salamat region of Chad. Following the identification of this cluster of positive HEV patients, MSF in collaboration with the MoH quickly established a community based surveillance network and initiated water and hygiene interventions including controlled bucket chlorination, hygiene promotion and hygiene kit distribution.

We report on the epidemiological and laboratory findings of an outbreak of HEV in the town of Am Timan, following its detection in September 2016 to April 2017. In addition, we conducted a case-test-negative study [14] to identify possible risk factors associated with being a confirmed case of HEV to inform future outbreak responses.

## Methods

### Case definitions

We defined suspected cases as those presenting with AJS at the hospital or in the community, in the region of Salamat from epidemiological week 33, 2016, onwards. AJS was defined as a person presenting with “yellow eyes”. This sensitive case definition has shown to be useful in previous outbreaks of HEV as it can be easily explained and non-medical persons can be trained on it easily. Confirmed cases were defined as suspected cases testing RDT positive for HEV. Discarded cases were suspected cases testing RDT negative for HEV.

### Case finding and data collection

Active case finding was organised through a community based surveillance network, which used 160 community health workers to conduct house-to-house visits (on a bi-weekly basis) to screen for AJS and refer persons to hospital with AJS at risk for clinical complications (pregnant women, children <1 year of age and persons with AJS who were vomiting and/or had altered mental status). Community health workers filled in a structured paper-based surveillance questionnaire for each AJS case identified which collected information on demographics, household characteristics, water sources used, pregnancy status and symptoms.

All hospital referrals (and self-referred individuals) underwent a medical assessment to determine severity of illness at the hospital. AJS cases presenting at the hospital that were pregnant or severely ill were tested for HEV infection using Assure^®^ IgM HEV RDTs. Health workers at the hospital also filled in a structured medical questionnaire for each AJS case.

As the surveillance and medical questionnaires were used for active surveillance in the outbreak, they were piloted and improved at the start of surveillance activities and immediately implemented for routine use. The collected data in the questionnaires was entered into an Excel datasheet by a data-entry clerk using unique identifiers for each case.

### Laboratory investigation

In October 2016, blood specimens were sent to Sanquin Laboratories in Amsterdam, the Netherlands, and were tested for anti-HEV antibodies using the Wantai enzyme-linked immunosorbent assay (EIA) and for HEV RNA using polymerase chain reaction (PCR). Genotyping was also performed on HEV PCR positive samples. Differential diagnoses for malaria, hepatitis A, hepatitis B, hepatitis C, yellow fever, zika, dengue, west nile, rift valley and chikungunya were performed using EIAs.

### Descriptive epidemiology

We calculated proportions for epidemiological characteristics by case definition category. We compared the differences in proportions between categorical variables using chi-squared tests.

### Case test negative study

Using surveillance data, we compared epidemiological characteristics of cases (confirmed cases) with controls (discarded cases) by calculating unadjusted odds ratios (ORs) and their respective 95% confidence intervals (95%CI). Risk factors were considered to be associated with being a confirmed case of hepatitis E if the p-value of the association was <0.05. We conducted stratified analyses to exclude relevant confounding and effect modification. Multivariable logistic regression analysis was also conducted but has not been presented due to the small sample size of our data and a lack of plausible reasoning for the variables of interest to influence association.

### Ethics

The Operational Centre Amsterdam Medical Director exempted this study from full review in accordance with the MSF Ethical Review Board guidelines, as it represented routine program monitoring and evaluation related work in the context of outbreak response where respondents were not exposed to risks. All data was anonymised for analysis and reported accordingly.

## Results

### Descriptive epidemiology

**Fig 1:**
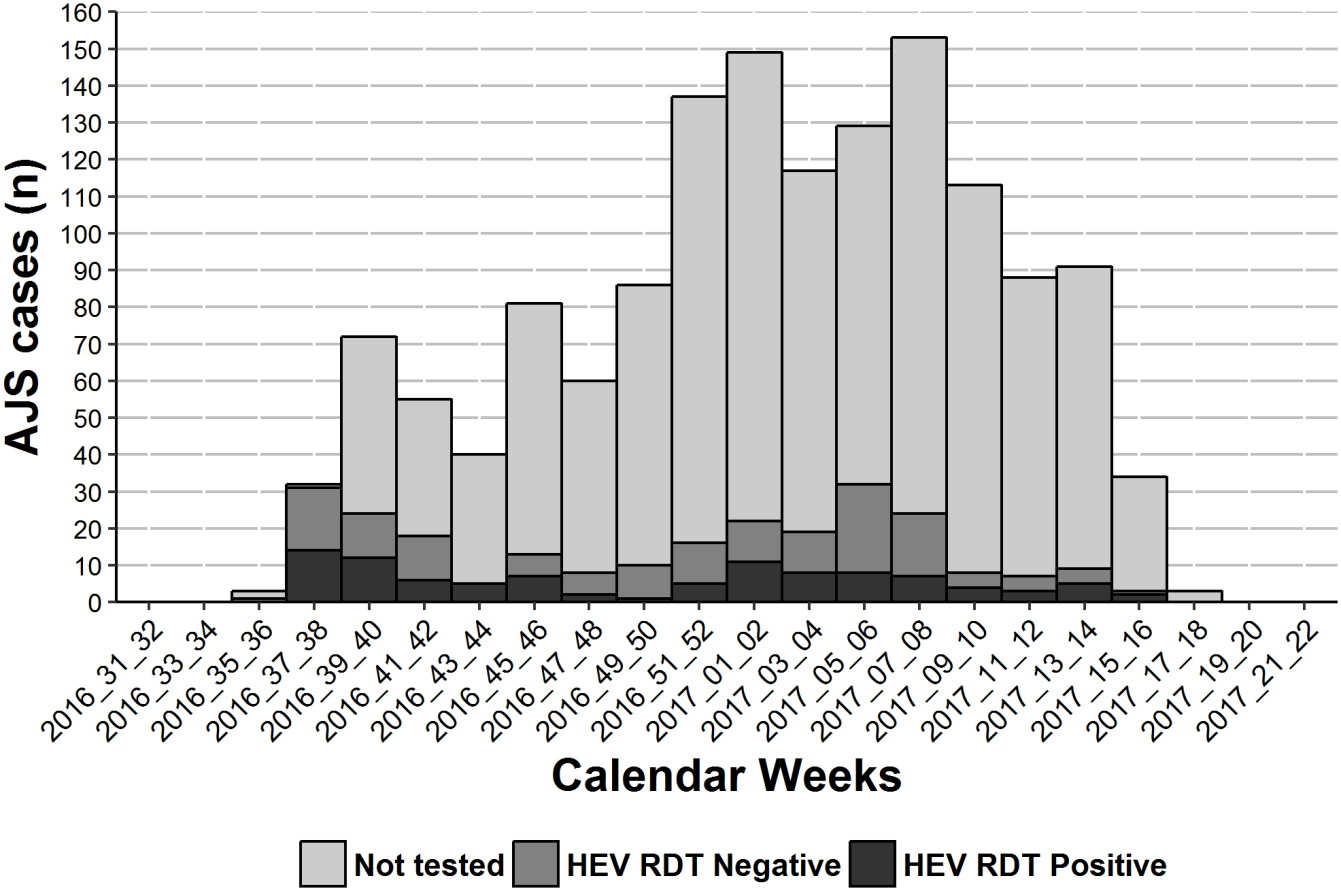
Epidemic curve of suspected cases, confirmed HEV cases and discarded cases on bi-weekly epidemiological week periods. **Am Timan, Chad, 2016–2017** (suspected cases = 1193; discarded cases = 150, confirmed cases = 100).

Between week 33/2016 and week 18/2017, 1443 suspected cases were identified. Cases peaked between week 51/2016 and week 8/2017, reaching 153 in weeks 7 and 8/2017. Of these 1443 suspected cases, 1193 (83%) were not tested; 250 (17%) were tested with a HEV RDT of which 100 (40%) were positive (confirmed) and 150 (60%) were negative (discarded) (Fig 1). The cumulative attack rate was 2% (1293 confirmed and suspected AJS cases in estimated 65,000 population).

The majority, 59% (n = 59) of confirmed cases were female compared to 47% (n = 562) of the suspected cases were females (p = 0.029) (Table 1). Confirmed cases were older; 62% (n = 61) of confirmed cases versus 42% (n = 503; p<0.001) of suspected cases belonged to the 15–44 year old age group.

**Table 1:** Characteristics of cases of acute jaundice syndrome and those testing positive and negative for HEV using RDTs RDTs during an acute outbreak of HEV in Am Timan, Chad, 2016–2017 (suspected cases = 1193; confirmed cases = 100; discarded cases = 150).

Characteristic	Suspected cases			Confirmed cases			Discarded cases			P-valueⱡ
	n	N	%	N	N	%	n	N	%	
**Sex**										
Male	631	1193	53	41	100	41	55	150	37	0.029
**Age group (years)**										
0 to 4	153	1187	13	11	99	11	11	149	7	0.724
5 to 14	478	1187	40	23	99	23	32	149	21	0.001
15 to 44	503	1187	42	61	99	62	99	149	66	<0.001
≥44	53	1187	4	4	99	4	7	149	5	1.000
**Household make up**										
≥5 persons in household	859	1020	84	47	53	89	84	108	78	0.497
≥ 2 children in household	628	1008	62	37	53	70	55	105	52	0.339
≥ 5 children in household	47	1008	5	6	53	11	1	105	1	0.065
Other household member with jaundice	131	1014	13	10	58	17	9	111	8	0.455
**Pregnant or post-partum**										
Pregnant	16	42	38	15	29	52	33	59	56	0.371
Post-partum	1	8	12	2	16	12	1	23	4	1.000
**Clinical status**										
Hospitalised	4	544	1	44	96	46	38	143	27	<0.001
Fever	525	575	91	64	92	70	88	135	65	<0.001
Nausea	308	572	54	53	93	57	71	137	52	0.651
Vomiting	590	703	84	57	65	88	72	104	69	0.536
Epigastric pain	348	571	61	54	91	59	74	137	54	0.861
Itching	405	569	71	51	91	56	62	138	45	0.005
Diarrhoea	85	563	15	12	93	13	17	138	12	0.693
Abnormal mental state	2	524	0	11	94	12	8	136	6	<0.001
**Neighbourhood of residence**										
Anfandock	38	1181	3	5	96	5	7	149	5	0.456
Ganatir	208	1193	17	7	100	7	27	150	18	0.011
Ridina	135	1181	11	8	96	8	4	149	3	0.449
Riad	76	1181	6	7	96	7	3	149	2	0.911
Taradona (all)	315	1193	26	19	100	19	33	150	22	0.132

^ⱡ^ P-values derived from Pearson’s chi-squared test between suspected and confirmed cases

Amongst the 716 female cases reported, 562 were suspected cases (47%) and 59 were confirmed (8.2%). Eighty six women out of the 621 suspected and confirmed cases (13.8%) self-reported as being pregnant. For 31 pregnant women, a medical assessment was completed and out of these for four women we were able to document the outcome of their pregnancy during their hospitalisation. Two women experienced a spontaneous abortion (both in their second trimester and both confirmed HEV cases) and two women delivered a surviving baby (both in their third trimester, one confirmed HEV case).

For 1020 (71%) suspected cases and 53 (53%) confirmed cases, we had information on household makeup. Household makeup did not differ between suspected cases and confirmed cases with 89% (n = 47) of households among confirmed cases having > = 5 persons living in the household. Ten of 58 (17%) confirmed cases had another household member with jaundice, similar to suspected cases.

The proportion of pregnant and post-partum women were similar among suspected and confirmed cases; with 52% (n = 15) and 12% (n = 2) of female confirmed cases being pregnant and post-partum, respectively.

The most common clinical symptoms reported amongst confirmed cases with available information were vomiting (n = 57, 88%), fever (n = 64, 70%), epigastric pain (n = 54, 59%). Fewer confirmed cases reported fever compared to suspected cases (n = 64, 70% versus n = 525, 91%; p<0.001). Also more confirmed cases reported an abnormal mental state (n = 11, 12% versus n = 2, 0%, p<0.001) than suspected cases.

**Fig 2:**
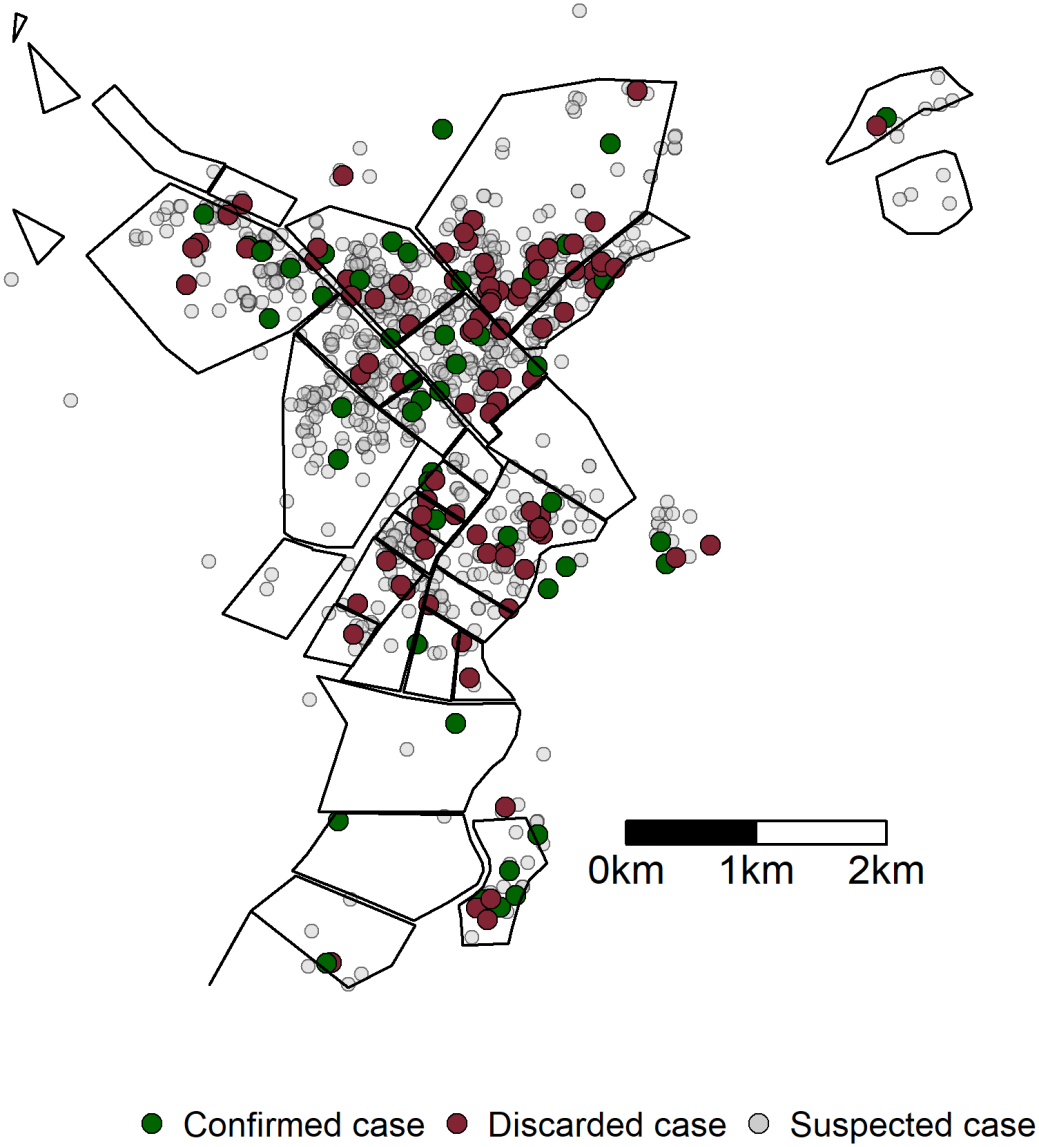
Geospatial distribution of suspected cases, confirmed HEV cases and discarded cases during an outbreak in Am Timan, Chad, 2016–2017 (suspected cases = 1193; discarded cases = 150, confirmed cases = 100).

The highest proportions of suspected and confirmed cases were reported from the districts of Taradona, with 26% (n = 315) and 19% (n = 19), respectively. The proportion of confirmed cases (n = 7, 7%) reported from Ganatir was significantly less than suspected cases (n = 208, 17%; p = 0.011) (Fig 2).

Forty-eight (8%), suspected and confirmed cases with information available were hospitalised; of these 44 (92%) were also confirmed cases. For 39 of the hospitalised patients (81%) a clinical outcome was recorded; 9 patients died (case fatality ratio hospitalised patients = 23%), 28 were discharged and two were lost to follow up. Amongst the hospitalised patients, there were 15 pregnant women, of whom three died (case fatality ratio = 20%).

Blood specimens of 37 confirmed and suspected cases were tested for anti-HEV antibodies in Sanquin Laboratories in Amsterdam. Of the specimens tested, 35 (95%) were positive for anti-HEV IgM of which 24 (68%) were also anti-HEV IgG positive. Eleven IgM positive specimens were tested for HEV RNA using polymerase chain reaction (PCR); all were positive. The genotype of the virus identified was Genotype 1e and was homologous to the HEV isolates from outbreaks in Sudan [15].

There was no difference in the proportion of confirmed and discarded cases that tested positive for malaria (31/96; 41.9% vs. 29/142; 34.9%; p = 0.4). More discarded cases tested positive for hepatitis B infection (17/142, 12%) compared to confirmed cases (3/96, 3.1%)(p = 0.02). Two discarded cases also tested positive for hepatitis C, two tested positive for west Nile virus infection and one for rift valley fever infection. This left 108 (72%) discarded cases with no alternative aetiology for AJS. No confirmed cases tested positive for other infectious disease aetiologies.

### Case test negative study

We included 100 confirmed cases and 150 discarded cases (controls) in the analysis. We found no significant differences between cases and controls based on age or sex (Table 2). However, cases were more likely to live in households with more than two (OR 2.1, 95%CI 1.1–4.3) or more than five (OR 13, 95%CI 2.2–254) children under the age of 5 years compared with controls. Cases were also more likely, though not significantly, to have another household member with jaundice (OR 2.4, 95%CI 0.90–6.3) compared to controls.

**Table 2:** Risk factors for confirmed HEV infection with RDTs during an acute outbreak of HEV in Am Timan, Chad, 2016–2017.

Characteristic	Confirmed cases			Discarded cases			OR	95%CI	P-value
	n	N	%	n	N	%			
**Sex**									
Male	41	100	41	55	150	37	1.2	0.71–2.02	0.490
**Age group (years)**									
0 to 4	11	99	11	11	149	7	1.6	0.65–3.8	0.315
5 to 14	23	99	23	32	149	21	1.1	0.60–2.0	0.745
15 to 44	61	99	62	99	149	66	0.81	0.48–1.4	0.437
≥44	4	99	4	7	149	5	0.85	0.22–2.9	0.806
**Household make up**									
≥5 persons in household	47	53	89	84	108	78	2.2	0.9–6.4	0.101
≥ 2 children in household	37	53	70	55	105	52	2.1	1.1–4.3	0.038
≥ 5 children in household	6	53	11	1	105	1	13	2.2–254	0.018
Other household member with jaundice	10	58	17	9	111	8	2.4	0.90–6.3	0.081
**Pregnant or post-partum**									
Pregnant	15	29	52	33	59	56	0.84	0.35–2.1	0.710
Post-partum	2	16	13	1	23	4	3.1	0.28–71	0.368
**Clinical status**									
Hospitalised	44	96	46	38	143	27	2.3	1.4–4.1	0.002
Fever	64	92	70	88	135	65	1.2	0.69–2.2	0.491
Nausea	53	93	57	71	137	52	1.2	0.73–2.1	0.441
Vomiting	57	65	88	72	104	69	3.2	1.4–7.9	0.008
Epigastric pain	54	91	59	74	137	54	1.2	0.73–2.1	0.428
Itching	51	91	56	62	138	45	1.6	0.92–2.7	0.100
Diarrhoea	12	93	13	17	138	12	1.1	0.47–2.3	0.895
Abnormal mental state	11	94	12	8	136	6	2.1	0.82–5.7	0.122
**Water point use**									
City water	11	43	26	17	85	20	1.4	0.57–3.25	0.472
Borehole	11	39	28	17	84	20	1.4	0.57–3.25	0.472
Communal tap	1	38	3	9	81	11	0.22	0.01–1.23	0.157
River	2	43	5	6	85	7	0.69	0.1–3.19	0.665
**Handwashing practices**									
Always washing hands before eating (compared to sometimes washing hands)	29	36	81	75	81	93	0.33	0.1–1.1	0.065
Soap available for handwashing	14	35	40	33	81	41	0.97	0.43–2.2	0.941
**Neighbourhood of residence**									
Riad	7	96	7	3	149	2	3.8	1.0–18	0.056
Ridina	8	96	8	4	149	3	3.3	1.0–12.6	0.057
Ganatir	7	100	7	27	150	18	0.34	0.13–0.78	0.016

Cases were more likely to present with vomiting (OR 3.2, 9%CI 1.4–7.9) and be hospitalised (OR 2.3, 95%CI 1.4–4.1) than controls. Stratified analyses suggested vomiting could be a negative confounder for the association between hospitalisation and being a case (hospitalisation adjusted OR: 4.7, 95%CI: 2.3–10, p < 0.001).

Water sources used by cases and controls and the availability of soap at the household level were similar for cases and controls. Cases were less likely to report always washing hands before meals compared with controls (OR 0.33, 95%CI 0.1–1.1), but this association was not statistically significant.

Compared with controls, cases were more likely to live in the neighbourhoods of Riad (OR 3.8, 95%CI 1.0–1.8) or Ridina (OR 3.3, 95%CI 1.0–12.6), in north-western Am Timan (though not statistically significant), but were significantly less likely to live in the neighbourhood of Ganatir (OR 0.34, 95%CI 0.13–0.78).

## Discussion

This HEV outbreak of genotype 1e in Chad was the largest HEV outbreak in sub-Saharan Africa that has been detected in a setting that was not a refugee camp. The magnitude of the outbreak was smaller than those reported from Uganda 2007 and South Sudan in 2006 [2,16]. However, the clinical characteristics and case fatality of hospitalised and confirmed cases remained similar to those reported from other outbreaks. Even though the overall impact of this outbreak in an urban setting was limited and few deaths were reported, the observed clinical manifestations of HEV were similar to those seen in refugee and IDP camps.

The epidemic curve suggested a point source followed by continuous person-to-person transmission. However, despite the six week cycles, the successive waves of AJS likely resulted from the temporary stop on active case finding during week 44, 2016 due to a mass distribution of hygiene kits to all households in the town and periodic interruption of active case finding to undertake a mortality survey. The epidemic curve does nevertheless mirror the shape described in previous outbreaks [17–19].

The differences between suspected and confirmed cases suggest there were some biases to being tested for hepatitis E. The higher age of confirmed cases compared to that of suspected cases is also not unexpected as this has been determined in previous outbreaks [19,20]. The higher proportion of female confirmed cases compared to suspected cases is likely due to the targeted testing criteria for HEV in Am Timan during this outbreak for pregnant women with AJS. The observed difference between suspected and confirmed cases in presenting with fever and altered mental status may be due to the differences in self-reporting in the community versus being seen by medical professionals at the hospital; all confirmed cases went to the hospital. There is no clear explanation for why the proportion of confirmed cases coming from the neighbourhood in Ganatir would be lower than the suspected cases, but could suggest a different level of HEV transmission in this neighbourhood compared to the other neighbourhoods. Anecdotally, Riad and Ridina are considered to be the poorer neighbourhoods of Am Timan with limited sanitation infrastructure, which could explain a more efficient transmission of the virus.

Over half of tested cases were negative for HEV during this outbreak, with no other infectious aetiology found. In contrast, other types of hepatitis and malaria infection accounted for AJS cases detected during a large-scale outbreak in Uganda[21]. In Am Timan, the additionally detected AJS cases might be due to other reasons (anemia, nutritional deficiencies or other) of which we were unable to elucidate the cause [22]. Another possible reason for the high proportion of negatives could be linked to RDT performance, where PCR is the gold standard unfeasible for the setting [23,24].

We were unable to identify a single source of this outbreak. However, the epidemiological evidence suggests that, also in this HEV outbreak, transmission at household level played a role in its propagation as having another person with jaundice in the household and having more than five people in the household were predictive of being a confirmed case [10].

The higher risk of hospitalisation and vomiting among cases could be attributed to the clinical evolution of the disease, in that HEV infected persons HEV may present with more severe illness compared to those with jaundice from other causes. Alternately, it would suggest that confirmed cases were more encouraged to be hospitalised, but we do not have any information that supports that hypothesis. Nevertheless the confounding of hospitalisation by vomiting does support the notion that there was selective admission to hospital during this outbreak. This also explains why vomiting and hospitalisation as independently associated with being a case.

The possible role that small children play in the transmission of the virus is suggested in our findings and was already suggested in previous sero-prevalence studies for HEV during outbreaks in Uganda [25]. The role that behaviour around hygienic practices play in HEV transmission is also alluded to in our results in that people with poor self-reported handwashing practices were more likely to be confirmed cases. Thus, the continued need for targeted water, sanitation (latrine construction) and hygiene (health promotion and education around hygiene practices) interventions at household level during outbreaks with this virus is reaffirmed. The need for these interventions to also reach children is highlighted by our study, given their potential to act as spreaders of infection. These household characteristics (including children) have been described as risk factors in previous HEV outbreaks[17,25] as well as the storage of drinking water in large, open-mouthed containers and the sharing of basins for handwashing [2]. The sharing of handwashing basins as a risk factor is an interesting contrast to our findings and may be suggestive of differences in the baseline level of hygiene practices; furthermore in the setting of Am Timan, few people had basins and washed hands using a kettle for pouring water.

Limitations to active case finding, including periodic interruptions and the mobility of parts of the town population (semi-nomadic herders), suggests that detected AJS cases may be an underestimation of the true number. Three further limitations to the case test-negative study must be highlighted. First, several characteristics were self-reported and could thus have suffered from recall and social desirability bias. Second, due to small numbers, no multivariable logistic regression models could be constructed to take confounding or effect modification into consideration for the risk factor analysis. Finally, only a small proportion of AJS cases were eligible for testing in this outbreak response according to very strict criteria. Thus confirmed cases may not be representative of the AJS cases and controls might not be representative of the affected population. In addition, not all tested individuals had information on exposures available.

Despite the potential under-ascertainment of cases, the cumulative attack rate of 2% suggests the limited extent of this outbreak in Am Timan when compared to previous outbreaks with attack rates of 25% [18], 7.7 [10] and 6.3% [26]. Genotype 1e has been identified in larger and more severe outbreaks of HEV (in terms of case numbers and deaths)[27]. Therefore, the impact and geospatial extension of this HEV outbreak in Am Timan cannot be attributed to a less pathogenic virus. Thus, the only remaining hypothesis is that a proportion of the population in Am Timan was already immune to HEV when the current outbreak started. For this reason, a cross-sectional study is currently ongoing in Am Timan to estimate the seroprevalence of HEV and to better understand the role that children might play in the transmission of this virus at a community level.

## Conclusions

Our study suggests that HEV outbreaks in an open setting, while presenting similarly in clinical characteristics and case fatality, may be of lesser magnitude than in Sub-Saharan African refugee camp settings. Further, our study suggests household factors and area of residence (possibly linked to access to water and sanitation) play a role in HEV transmission, underscoring the need for future HEV outbreak responses to incorporate timely water and hygiene interventions and targeted hygiene promotion activities in programming.

## Supporting information

S1 File Surveillance and medical assessment questionnaires in French. (DOCX)

S2 File Surveillance and medical assessment questionnaires in English. (DOCX)
