# Supplementary material for: A large outbreak of Hepatitis E virus genotype 1 infection in an urban setting in Chad likely linked to household level transmission factors, 2016-2017
Source: PLoS One. 2017 Nov 27;12(11):e0188240. doi: 10.1371/journal.pone.0188240 (PMC5703542; doi:10.1371/journal.pone.0188240)
Supplement: S1 File — (DOCX) [file pone.0188240.s001.docx]

**MSF-OCA Am Timan –**

**Numéro de cas: ______________**

**Questionnaire de Surveillance des Cas Hépatite E**

**Date:** ____________________ **Enquêteur:** _______________________ Encodé par : ______________________

**Eq :_­­­_____AC :_____Bloc :__________Men :_________**

1. Référé à votre équipe par :  Hôpital  Superv. AC

2. Quartier/Village:______________________________

3. GPS de Maison:

Latitude : _____________ Longitude : ______________

**Information sur la personne interrogée:**

4. Statut Interrogée ?: …………….. Patient  Parent

5. Si parent, sa/son nom?:________________________

6. Téléphone: __________________________________

7. Nombre de personnes dans le ménage : ___________

1. Nombre masculin: ________________________
2. Nombre feminin: ________________________
3. Nombres d’enfants <5ans :_________________

**Information sur le patient:**

8. Nom: _______________________________________

9. Sexe:…………………..…………………………. M  F

10. Age: ___________________________________ ans

11. Référé à l’hôpital par Agent Com?  Oui  Non

1. Si oui, le patient est allé ?  Oui  Non

12. *Est-ce que le patient est présent*?  Oui  Non

1. Si « Oui » --> Continuer l’enquête à question 13.
2. SI « Non »--> Sauter à question 16.

13. *Est-ce que le patient a la jaunisse*?  Oui  Non

14. Est-ce que le patient a les symptômes suivants ?.......

1. Vomissements ?  Oui  Non
2. Etat mental altéré?  Oui  Non
3. Enceinte?  Oui  Non
4. Agée moins d’un an ?  Oui  Non

15. *Allez-vous référer à l’hôpital* ?  Oui  Non

16. Est-ce qu’il y a d’autre personne avec jaunisse dans le ménage?..........................................  Oui  Non

1. Si « Oui », combien? ______________________

(*Pour chaque nouveau cas, il faut compléter une autre fiche)*

**Questions concernant l’accès à l’eau:**

17. Est-ce que vous utilisez les points d’eau suivants ?

*Demander « Oui/Non » pour chaque option :*

1. Forage ?  Oui  Non
2. Bonne Fontaine ?  Oui  Non
3. Rivière?  Oui  Non
4. Eau de la ville ?  Oui  Non
5. Autre ?  Oui  Non

Si autre :*_____________________________*

18. Quel point utilisez-vous le plus souvent ?_________

19. Est-ce que vous utilisez le stockage suivant ?.............

*Demander « Oui/Non » pour chaque option :*

1. Jarre ?  Oui  Non
2. Jerrycan ?  Oui  Non
3. Fût ?  Oui  Non
4. Autre ?  Oui  Non

Si autre :________________________________

20. La dernière fois que vous avez cherché de l’eau, quel type de stockage avez-vous utilisé ?_________________

21. Quelqu’un a ajouté du chlore la dernière fois que vous avez cherché de l’eau ?  Oui  Non

22. « Pouvez-vous me montrer votre stockage ?»

1. *Combien de stockage ont-ils ?__________________*
2. *Est-ce qu’on pourrait entrer la main dans le stockage et contacter l’eau*?  Oui  Non

**Observation Sanitaire et Environnemental :**

23. Est-ce que vous vous lavez les mains avant de manger ? *Lire les options suivantes :*

Toujours  Quelquefois  Jamais

24. «Pouvez-vous me montrer ou vous lavez vos mains?»

*Est-ce qu’il y a de l’eau*?  Oui  Non

*Est-ce qu’il y a du savon*?  Oui  Non

25. Quel type de défécation avez-vous utilisé pendant la dernière semaine? *Demander pour chaque option :*

1. Rivière  Oui  Non
2. A l’air libre ?  Oui  Non
3. Latrine ?  Oui  Non

*Si le répondent dit «Oui » pour Latrine, demander :*

26. Combien de personnes utilisent ce latrine ? _______

**MSF-OCA Am Timan –**

**Numéro de cas: ______________**

**Questionnaire Médical des Cas Hépatite E**

1. **Date:** ____________________ **Enquêteur:** _______________________
2. Numéro ID de clinique/hôpital:_____________________
3. Nom de patient : _____________________________________
4. Téléphone :___________________________________________
5. Quartier: ______________________________________________
6. Age : _______________________________  Ans Mois
7. Sexe :…….... ………………………………. M  F
8. Il/elle est déjà évalué pour la jaunisse (à l’hôpital, communauté, ou référé) ? ……….. Oui  Non

Si « Oui », l’ancien numéro de cas :___________________

1. Est-ce que le patient était référé par l’Agent Communautaire de MSF ?.. ……….. Oui Non

Si « Oui », numéro de cas : __________________________

1. Est-ce qu’il/elle a la jaunisse ?  Oui  Non
2. Date de début de la jaunisse ? ______________________
3. Est-ce que le patient a les symptômes suivants ?...
4. Fièvre ?............... ………………….  Oui Non
5. Nausées/Anorexie ?...... ………. Oui Non
6. Vomissements ?.............. ………. Oui Non
7. Douleur épigastrique ? ………. Oui Non
8. Démangeaisons ?........... ………. Oui Non
9. Mal de tête ?..... ………….………. Oui Non
10. Arthralgies ?..... ………….………. Oui Non
11. Diarrhées ?........ ………….………. Oui Non
12. Saignements ?.. ………….………. Oui Non

**Exam** **Médical**:

1. Fièvre ≥ 38.0°C?...... ………….………. Oui Non
2. Etat mental :  Normal  Confus/somnolent

Coma  Inconnu

1. Est-ce qu’il y a d’autre personne avec jaunisse dans le ménage?............................................  Oui Non
   1. Si « Oui », combien? ______________________________
   2. Numéro de cas pour chacun : ____________________
2. La patiente est-elle enceinte ?........ Oui  Non

Si oui, trimestre?.... 1  2  3  Inconnu

1. Postpartum?.....……………………….... Oui  Non
   1. Combien de jours après l’accouchement ? _______
   2. Résultat de la Grossesse**:**

Sortie, enceinte

Avortement/mort-né

Naissance, enfant toujours vivant

Naissance, enfant décédé

Décès maternelle

(Si oui, la date de décès :____________________)

Inconnu/Introuvable

**Laboratoire :**

1. Prélèvements du sang ?…...……….. Oui  Non

Si «  Oui », date de prélèvement:____________________

1. Résultats de prélèvements :
2. Malaria RDT :  Pos  Neg
3. Hépatite B :  Pos  Neg
4. Hépatite C :  Pos  Neg
5. Hépatite E :  Pos  Neg

**Hospitalisation :**

1. Le patient était hospitalisé ?......... Oui Non

Si oui, date d’hospitalisation : ______________________

1. **Résultat Clinique**:

Déchargé/Guéri

Référé

Echappé

Décédé

(Si oui, la date de décès :____________________)

Inconnu/Introuvable
