# Supplementary material for: A large outbreak of Hepatitis E virus genotype 1 infection in an urban setting in Chad likely linked to household level transmission factors, 2016-2017
Source: PLoS One. 2017 Nov 27;12(11):e0188240. doi: 10.1371/journal.pone.0188240 (PMC5703542; doi:10.1371/journal.pone.0188240)
Supplement: S2 File — (DOCX) [file pone.0188240.s002.docx]

**MSF-OCA Am Timan –**

**Case number: ______________**

**Surveillance Questionnaire Hepatitis E Cases**

**Date:** ____________________ **Interviewer:** _______________________ Entered by : _____________________

**Team :_­­CHW :_____Bloc k:__________HH :_________**

1. Referred to your team by :  Hospital  Outreach Supervisor

2. Quartier/Village:______________________________

3. GPS of HH:

Latitude : _____________ Longitude : ______________

**Interviewee information:**

4Status ?: …………….. Patient  Family member

5. Si family member, his/her name?:_______________

6. Phone number:______________________________

7. Number of persons in HH: ___________

1. Number male: ________________________
2. Number female: ________________________
3. Number children <5 years :_________________

**Information about the patient:**

8. Name:______________________________________

9. Sex:…………………..…………………………. M  F

10. Age: ___________________________________ yrs

11. Referred to Hospital by CHW?  Yes  No

1. If yes, did the patient go ?  Yes  No

12. *Is the patient present*?  Yes  No

1. If « Yes » --> Go to question 13.
2. If « No »--> Go to question 16.

13. *Is the patient jaundiced*?  Yes  No

14. Does the patient have the following symptoms ?.......

1. Vomiting ?  Yes  No
2. Altered mental state?  Yes  No
3. Pregnant?  Yes  No Less than 1 year of age ?  Yes  No

15. *Will you refer to the hospital* ?  Yes  No

16. Any other person with jaundice in the HH?..........................................  Yes  No

If «Yes », how many? ______________________

(*For each new case complete a seperate questionnaire)*

**Questions about access to water:**

17. Do you use any of the following water sources ?

*Ask for each type :*

1. Bore hole ?  Yes  No
2. Communal Tap ?  Yes  No
3. River?  Yes  No
4. City water ?  Yes  No
5. Other ?  Yes  No

Specify other :*__________________________*

18. Which source do you use the most often ?________

19. Do you use any of the following water storage ?.......

*Ask for each type :*

1. Ceramic jar ?  Yes  No
2. Jerrycan ?  Yes  No
3. Barrel ?  Yes  No
4. Other?  Yes  No

Specify others :_________________________

20. Which strorage did you use last time you collected water ?_________________

21. Last time you collected water did someone treat it with chlorine ?  Yes  No

22. « Can you show me your water storage ?»

1. *How many containers are there ?______________*
2. *Is it possible to put your hand inside the container*?  Yes  No

**Sanitation and environment observation :**

23. Do you wash your hands before eating ? *read the following options :*

Always  Sometimes  Never

24. «Can you show me where you wash your hands?»

*Is there water present*?  Yes  No

*Is there soap present*?  Yes  No

25. Where did you defecate during the last week? *Ask each item :*

1. River  Yes  No
2. Open air ?  Yes  No
3. Latrine ?  Yes  No

*If the respondent answered yes for latrine :*

26. How many people use this latrine ? _______

**MSF-OCA Am Timan –**

**Case number: ______________**

**Medical Questionnaire for Hepatitis E**

1. **Date:** ____________________ **Interviewer:** _______________________
2. Clinical ID number:_____________________
3. Patient name : _____________________________________
4. Phone :___________________________________________
5. Neighbourhood: _____________________________________
6. Age : ____________________________  Yrs Months
7. Sex :…….... ………………………………. M  F
8. Wash he/she already evaluated for jaundice (at the hospital, community level or refererred)? ……….. Yes  No

Si « Yes », old Case ID :___________________

1. Were they referred by a CHW ?.. Yes No

If « Yes », case ID : __________________________

1. Is the patient jaundiced ?  Yes  No
2. Date of start of jaundice ? ______________________
3. Does the patient have the following ?...
4. Fever ?............... ………………….  Yes No
5. Nausea/anorexia ?...... ………. Yes No
6. Vomiting?.............. ………. Yes No
7. Epigastric pain ? ………. Yes No
8. Itching ?........... ………….………. Yes No
9. Headache ?..... ………….………. Yes No
10. Arthralgias ?..... ………….………. Yes No
11. Diarrhea ?........ ………….………. Yes No
12. Bleeding ?.. ………….………. Yes No

**Medical Exam**:

1. Fever ≥ 38.0°C?...... ………….………. Yes No
2. Mental state :  Normal  Confused//sleepy

Coma  Unknown

1. Anyone else with jaundice in the HH?....................  Yes No
   1. If « Yes », how many?__________________________
   2. Hep E case number for each : __________________
2. Is the patient pregnant ?........ Yes  No

If yes, which trimester?.... 1  2  3

Unknown

1. Postpartum?.....……………………….... Yes  No
   1. How many days postpartum ? _______
   2. Pregnancy outcome**:**

Exited, still pregnant

Abortion/still birth

Birth, baby still alive

Birth, baby passed away

Maternal death

(date of death :____________________)

Unknown

**Laboratoire :**

1. Blood sample taken ?…...……….. Yes  No

If « Yes », date of sample:____________________

1. Sampling results :
2. Malaria RDT :  Pos  Neg
3. Hepatitis B :  Pos  Neg
4. Hepatitis C :  Pos  Neg
5. Hepatitis E :  Pos  Neg

**Hospitalisation :**

1. Is the patient hospitalised ?......... Yes No

If yes, date of hospitalisation : ______________________

1. **Clinical outcome**:

Discharged/cured

Referred

Lost to follow up

Died

(Date of death :____________________)

Unknown
